# FAM83B promotes the invasion of primary lung adenocarcinoma via PI3K/AKT/NF-κB pathway

**DOI:** 10.1186/s12890-022-02303-5

**Published:** 2023-01-23

**Authors:** Jing Zhang, Jiajia Wang, Ke Yue, Panpan Li, Wenping Shen, Xiaowen Qiao, Yan Wang, Xiaojuan Wu

**Affiliations:** 1 Department of Pathology, School of Basic Medical Sciences, Shandong University, Jinan, 250012 Shandong China; 2 Department of Pathology, Qilu Hospital, Shandong University, Jinan, 250012 Shandong China

**Keywords:** FAM83B, TIMP-1, Lung adenocarcinoma, Migration, Invasion

## Abstract

**Objects:**

The family with sequence similarity 83B (FAM83B) is one of the markers for poor prognosis in several carcinomas, but the expression and the mechanism resulted in malignant phenotype in lung adenocarcinoma (LUAD) remain to be elucidated.

**Methods:**

Data of RNA-seq in LUAD were downloaded from the cancer genome atlas (TCGA) database for differential expression and survival analysis, and immunohistochemistry was employed to analyze the protein expression of FAM83B in 126 cases of primary LUAD. The LUAD cell lines were collected for the detection of the effects on migration and invasion. Then, western blot was performed to measure the expression of tissue inhibitor of metalloproteinase (TIMP)-1 and activation of PI3K/AKT/NF-κB pathway.

**Results:**

FAM83B was overexpressed in multiple types of carcinomas; The differential expression analysis revealed that the level of FAM83B was higher in LUAD than that in para-carcinoma; The patients with overexpression of FAM83B were with shorter overall survival (OS), disease specific survival (DSS) and progress free interval (PFI); Enrichment analysis suggested it was related to the focal adhesion of LUAD. Immunohistochemistry analysis demonstrated that higher FAM83B expression was positively related to lymph node metastasis in primary. Scratch assay and Borden chamber assay showed that the overexpression of FAM83B promoted migration and invasion activity in vitro. Furthermore, high level of FAM83B accelerated the tumorigenesis in vivo. Western blot showed that TIMP-1 was upregulated in H1299/FAM83B OE cells accompanying by the activation of PI3K/AKT/NF-κB pathway.

**Conclusions:**

FAM83B was a marker for poor prognosis of LUAD and it might promote the expression of TIMP-1 by activating PI3K/AKT/NF-κB pathway and then affect the ECM balance, which resulted in the migration and invasion of LUAD.

**Supplementary Information:**

The online version contains supplementary material available at 10.1186/s12890-022-02303-5.

## Background

The recurrence and metastasis are barriers in the treatment of LUAD which will show stronger proliferation, migration and invasion activity and usually lead to the cancer-related mortality [1]. It is a key to explore potential therapeutic target which can predict the invasion and metastasis of LUAD.

Family with sequence similarity 83 (FAM83), including eight members (A-H), is conservatively expressed in vertebrates [2]. All the members have an unknown functional conserved domain with about 300 amino acids at the N-terminal (DUF1669 domain) which results in the different functions among all the family members. FAM83B is a member of FAM83 family. FAM83B overexpressed in several kinds of malignant tumors, such as breast cancer, cervical cancer, endometrial cancer, pancreatic ductal adenocarcinoma, etc. Overexpression of FAM83B could promote the malignant biological behaviors of these tumors [3–5]. It was reported that the lung squamous carcinoma with FAM83B over expression was usually with a shorter disease-free survival (DFS), but it could not affect the overall survival (OS). They also observed that the expression level was very low in LUAD. However, others reported that the over expression of FAM83B was negatively correlated with the survival in non-small cell lung carcinoma (NSCLC) patients and it was correlated with the activation of EGFR signal pathway and mutation of EGFR [6]. Yamaura et al. found that FAM83B was a potential therapeutic target for EGFR wild type (WT) in lung adenocarcinoma [7]. At present, the over expression of FAM83B is an important marker of poor prognosis in a variety of malignant tumors, but the role of FAM83B in LUAD remains to be fully elucidated [8].

In this study, we analyzed the differences expression of FAM83B between the primary LUAD and the para-carcinoma pulmonary tissue by analyzing The Cancer Genome Atlas (TCGA) and various public databases. Then, we evaluated the gene expression of FAM83B in carcinomas and the genes and functions related to co-expression of FAM83B through multidimensional analysis and discussed the possible mechanism of malignant phenotype induced by FAM83B in LUAD cells.

## Materials and methods

### TCGA database

TCGA contains 10,363 samples of 33 tumor types and 730 of para-cancer tissue samples. In this study, we used TCGA-pan carcinoma data to analyze the expression of FAM83B in different types of cancer; Then, we analyzed the RNA-sequence data included 535 tumor samples and 59 para-cancer tissues in LUAD. 526 samples were accompanied by survival outcomes in the database.

### LinkedOmics analysis

The LinkedOmics database (http://www.linkedomics.org/login. php) is a network-based platform, which provides a comprehensive multi-omics data analysis tool for TCGA database [9]. Pearson correlation coefficient was used for the statistical analysis of positive co-expression of FAM83B and displayed in the form of volcano map and heat map. The rank criterion was FDR < 0.05.

### R software

The Gene Ontology (GO) function and Kyoto Encyclopedia of Genes and Genomes (KEGG) (http://www.kegg.jp/) pathway enrichment of potential targets which may be positively related to FAM83B expression were analyzed using the cluster profiler software package of R. The ggplot2 software package was utilized to visualize the analysis data.

### Case series

We collected 126 primary lung adenocarcinoma and corresponding adjacent lung tissue samples of Qilu Hospital, Shandong University from January 2011 to December 2018. The above pathological samples were confirmed by clinicians. All cases were not treated before operation. Clinical-pathological features such as age at time of diagnosis, gender, smoking history, histological subtype, tumor size, lymph nodes involvement and TNM stage were collected.

### Immunohistochemistry

Tissue specimens were fixed in formalin and embedded in paraffin. Sections were autoclaved in EDTA buffer (pH 8.0) for antigen retrieval. After blocking in 10% goat serum, sections were incubated with a rabbit polyclonal anti‐FAM83B antibody (NBP1-86,764; Novus Biologicals, USA) with a dilution of 1:200 at 4 ℃ overnight. The slides were incubated in two-step plus Poly-HRP Anti- Mouse/Rabbit IgG Detection System (PV-9000, ZSGB-Bio, Beijing, China) following the manufacturer’s recommendations and visualized using DAB (ZSGB-Bio, Beijing, China) and then, rinsed in distilled water and counterstained with hematoxylin. The staining was evaluated under a microscope and positivity was judged when more than 10% of the area was occupied with moderate or strong positive cells.

### Cell lines culture

Human LUAD cell lines, H1299 and PC9/GR, were obtained from the American National Cancer Institute. Cells were maintained in RPMI medium1640, with [+] L-glutamine and [–] Phenol Red, supplemented with 10% (v/v) FBS, 100 lL/mL penicillin and 100 lL/mL streptomycin in humidity atmosphere with 5% CO_2_ at 37 °C. (Additional file 1).

### Lentiviral production and infection

Lentiviral particles carrying FAM83B cDNA and its flanking control (NC) were constructed by GeneChem (Shanghai GeneChem Co., Ltd., Shanghai, China). H1299 cells were infected with the lentiviral vectors and the cells named H1299/FAM83B OE and H1299/FAM83B NC with green fluorescent protein signals were selected for next experiments (Additional file 2: Fig. 1).

**Fig. 1:**
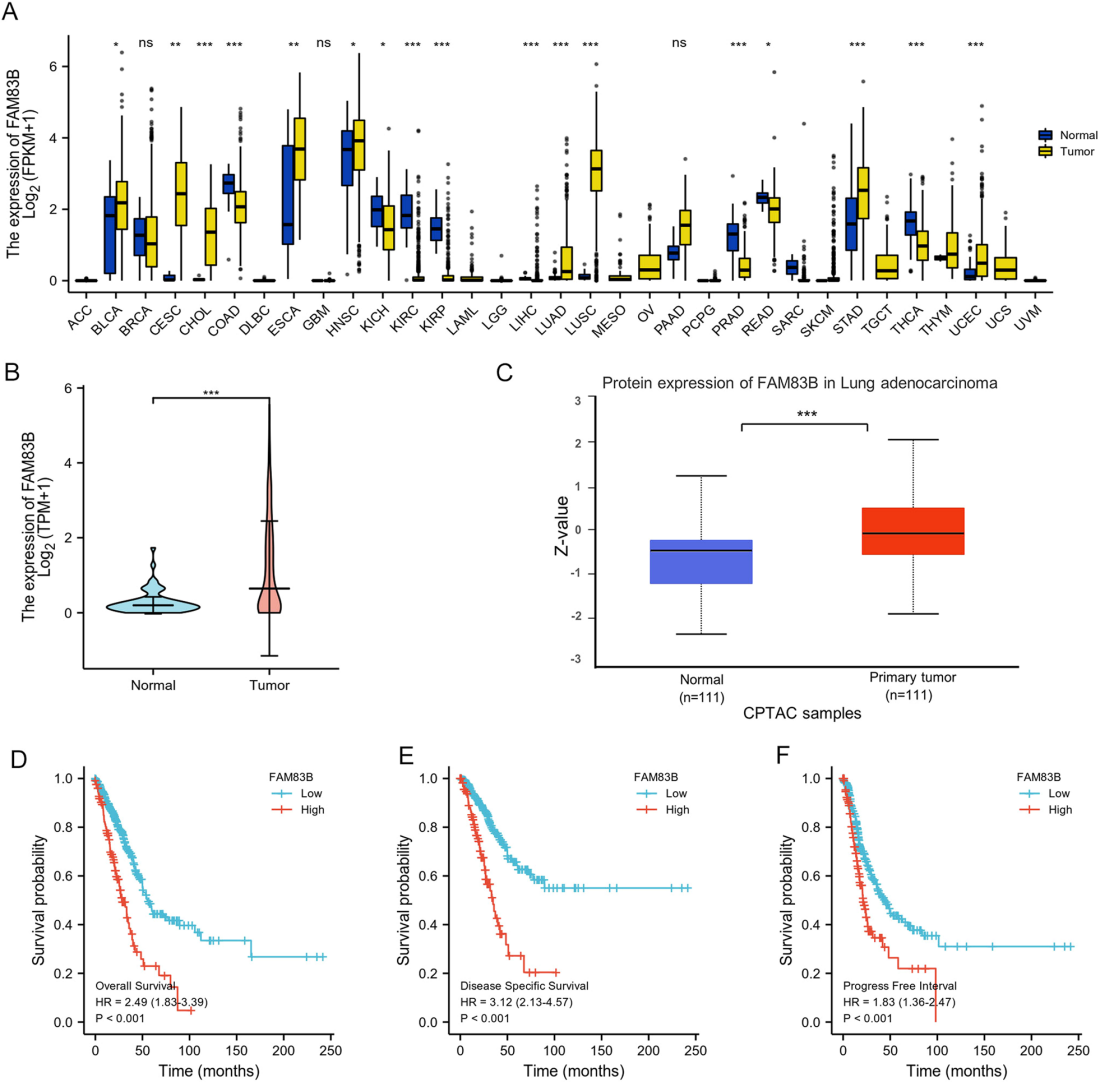
Expression of FAM83B in pan-carcinoma and primary LUAD. **A**: The TCGA database shows that FAM83B is upregulated in multiple types of carcinomas. **B**: The expression level of FAM83B mRNA between normal pulmonary tissues and LUAD. The Wilcoxon rank sum test showed that the unpaired sample of 59 normal tissues and 513 tumors showed that the expression of FAM83B in tumors were higher than those in normal samples and the median difference between the two groups was 0.824 (0.669–0.98) with statistical significance (*P* < 0.001); Among them, 57 paired samples showed that FAM83B in tumor was higher than that in normal lung tissue, and the median difference between the two groups was 0.582 (0.395–0.89), the difference was statistically significant (*P* < 0.001) **C**: The expression of FAM83B protein in primary LUAD was higher than that in the normal pulmonary tissues. **D**–**F**: Analysis of Kaplan Meier plot showed that the OS, DSS and PFI were significantly lower in high expression group than those in low expression queue. ***P < 0.001

### Transient transfection with siRNA to knockdown FAM83B gene (FAM83B siRNA) and its flanking control (siNC)

Three interference sequences si-1, si-2 and si-3 for FAM83B were designed and synthesized. The interference efficiency of si-2 is about 60%, which is significantly higher than that of si-1 and si-3 (Additional file 3: Fig. 2). Therefore, si-2 was further packaged as lentivirus vector. The FAM83B siRNA sequence was 5'-TTCGTTCCTCTTTAGTATT-3' which was designed and synthesized by GeneChem (Shanghai, China). PC9/GR cells were planted on a 6-well plate for 24 h before transfection (Additional file 2: Fig. 1). FAM83B siRNA and siNC were transfected into cells which were named PC9/GR FAM83B siRNA and PC9/GR FAM83B siNC using LipofectamineTM 2000 (Invitrogen Biotechnology, China), respectively. After 24-48 h, cells were collected for further experiments.

**Fig. 2:**
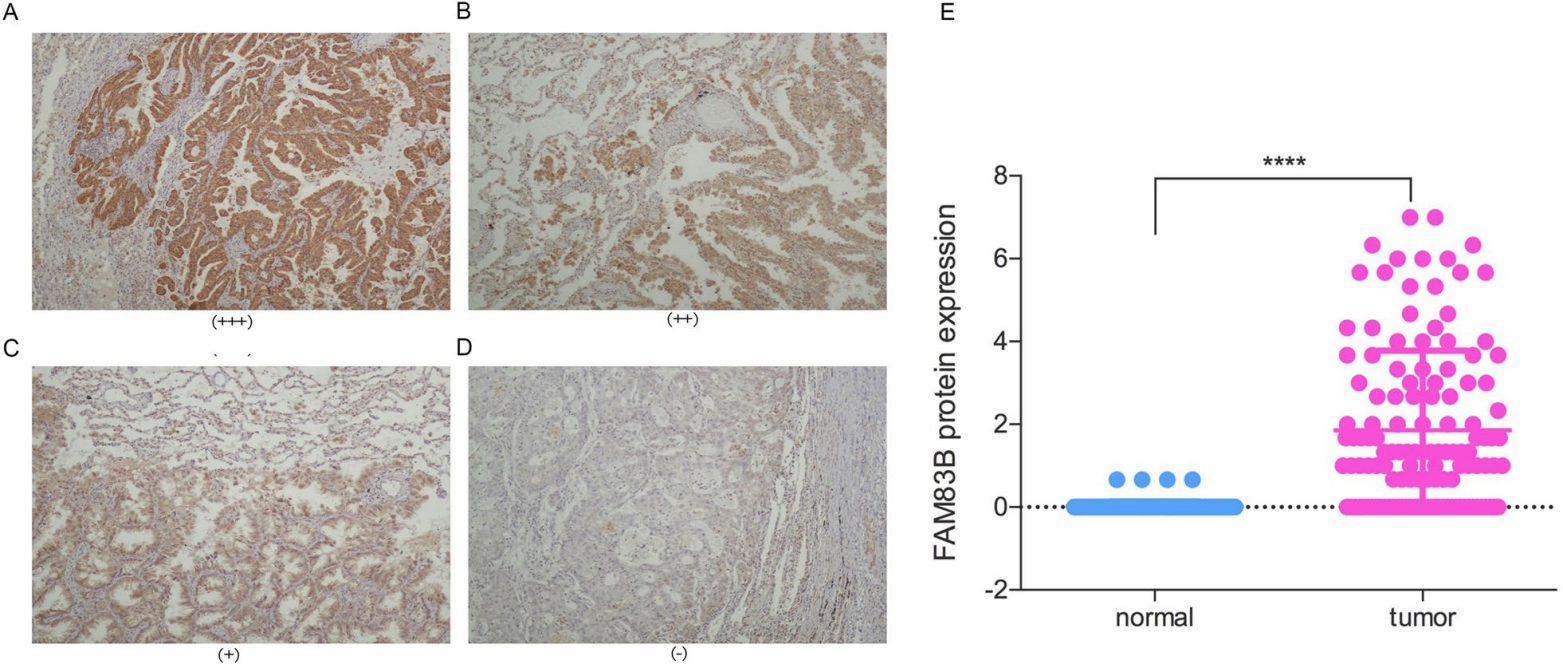
Analysis of the FAM83B expression in primary LUAD and para-carcinoma tissues by immunohistochemistry. **A–D**: A-D showed the strong positive, medium positive, positive and negative expression of FAM83B in primary LUAD, respectively. (200 × , 400 ×). **E**: The difference of FAM83B expression between para-carcinoma tissue and LUAD. ***P < 0.001

### Scratch migration and invasion assays

Cell migration was assessed using wound healing assay. Cell suspension was prepared and plated in 6-well plates, when it reached 100% confluency, scratch wounds were made using pipette tips and rinsed each well triple PBS washing. Then the cells were cultured in the medium containing 10% FBS. The cell motility images were photographed at 0, 24–36 h.

Invasion assays were carried out as described previously [10, 11]. Briefly, 5 × 10^4^ LUAD cells were suspended in the invasion medium and loaded into the upper compartment of Boyden chambers with 20 μg of BD MatrigelTM Basement Membrane Matrix (BD Biosciences, New Jersey, USA). Invasion medium was added to the lower compartment of the chambers. After the cells were cultured at 37 °C in a 5% CO_2_ atmosphere for 24 h, the filters were taken out from the chambers and the cells were then fixed with 4% paraformaldehyde and stained with 0.1% crystal violet dye solution. The cells were counted on 3 different fields on the down chamber. Samples were performed in triplicate.

### Quantitative real-time PCR (qRT-PCR)

All operations were followed the manufacturer’s protocol. Total RNA was extracted using TRIzol RNA extraction kit (Invitrogen, California, USA) and was reverse transcribed to cDNA using ReverTraAce qPCR RT Kit (Roche, Basel, Switzerland). Realtime PCR reactions were performed using the SYBR Green Master (Roche, Basel, Switzerland). The primers for FAM83B and GAPDH were as follows. FAM83B: forward, 5′- AGTTGACTGCCAGAAAGTGATG -3′ reverse, 5′- GATGCTAATGAAGACACCGAAT -3′.

GAPDH: forward, 5′- GGAGCGAGATCCCTCCAAAAT -3′ reverse, 5′-GGCTGTTGTCATACTTCTCATGG3′. All PCR experiments were repeated three times.

### Protein extraction and immunoblot analysis

Cell sediment was collected and lysed in ice-cold RIPA lysis buffer to obtain total protein. The protein concentration was determined by BCA protein assay kit (P0011, beyotime, Shanghai, China). Denatured proteins were separated by SDS-PAGE (10%) and transferred to polyvinylidene fluoride membranes (Millipore, Billerica, MA, USA). After blocking with 5% nonfat milk for 2 h, membranes were incubated at 4 °C overnight with primary antibodies: anti-FAM83B (NBP2-16,423; Novus;1:1000 dilution), anti-phosphorylated AKT(p-Akt1S473; CST, Shanghai, China; #4060; 1:1000 dilution), anti-AKT(CST; #2920; 1:1000 dilution), anti- matrix metalloproteinase(MMP)-9 (CST; #13,667; 1:1000 dilution), anti-TIMP-1(CST; #8946; 1:1000 dilution), anti-NF-κB p105/p50 (CST; #3035; 1:1000 dilution), anti-Tri-Methyl-Histone H3 (CST; #4909; 1:1000 dilution) and anti-GAPDH (GOODHERE, Hangzhou, China; AB-PR001; 1:1000dilution). Next, membranes were incubated with HRP-conjugated goat anti-rabbit IgG H&L secondary antibodies (Abways, Shanghai, China; AB0141; 1:50,000 dilution) for 30 min at 37 °C. Immunoreactivity was visualized using an enhanced chemiluminescence kit (Millipore, Darmstadt, Germany). ImageJ was used for quantitative analysis. Chemilumin Imaging System (Clinx science instruments Co.,Ltd, Shanghai, China) was used for chemiluminescence imaging. The gel exposure time was controlled between 2 and 10 s.

### Tumor xenograft in nude mice

4-week-old male BALB/c nude mice were randomly divided into two groups (*n* = 7/group). Stable H1299/FAM83B OE and H1299/FAM83B NC cells were collected and resuspended in physiological saline. 200ul cell suspension (about 1×10^8^ cells) was subcutaneously injected into the left armpit of nude mice, the tumor diameter was measured every 7 days. After 5 weeks, the tumor was observed by vivo fluorescence imaging system, then mice were euthanized by inhaling carbon dioxide and tumors were removed. The volume (V) = (length×width ^2^)/2 was calculated. All tumors for pathological examination were formalin-fixed, paraffin-embedded, then stained with H&E.

### Statistical analysis

All data were analyzed using GraphPad Prism version 6.0 and SPSS version 23.0. The Wilcoxon rank sum test was used to analyze the unpaired samples from TCGA database. The chi-square test and two-sided t test were used to analyze the correlation between FAM83B expression and corresponding clinic-pathological characteristics in primary lung adenocarcinoma patients. Log-rank test was applied to compare overall survival (OS), disease specific survival (DSS) and progress free interval (PFI) distribution. Significance identification included ns, *p* ≥ 0.05; *, *p* < 0.05; **, *p* < 0.01; ***, *p* < 0.001.

## Results

### The mRNA expression levels of FAM83B in human carcinomas

The mRNA sequencing results of 730 corresponding normal tissues and 10,363 different types of tumors were analyzed in TCGA database. The results showed that the levels of FAM83B in 10 types of cancers were higher than those in normal tissues, but in 7 kinds of cancers were lower than those in normal tissues with statistical significance. (Fig. 1A).

### The expression of FAM83B in LUAD

The mRNA sequencing results of the unpaired sample including 59 normal lung tissues and 535 LUAD samples were analyzed in TCGA database. The results showed the expression of FAM83B in tumors were higher than those in normal samples and the median difference between the two groups was 0.824 (0.669–0.98) with statistical significance (*p* < 0.001). (Fig. 1B). Furthermore, the results of protein expression showed the levels of FAM83B in LUAD were higher than those in normal tissues, using data from Clinical Proteomic Tumor Analysis Consortium (CPTAC) provided by UALCAN (Fig. 1C).

### The effect of FAM83B high expression on OS, DSS and PFI in LUAD

In the survival analysis, the follow-up periods of the patients varied between 25.4 to 39 months (median 26.9 months) in high expression group and between 50 to 105.6 months (median 57.5 months) in low expression group; And the OS durations were found to be significantly shorter in patients with FAM83B high expression compared to patients with FAM83B low expression (p < 0.001). In addition, the follow-up periods of the patients varied between 17.2 to 24.2 months (median 20.6 months) in high expression group and between 34.9 to 63.1 months (median 43.1 months) in low expression group; And the PFI durations were found to be shorter in patients with FAM83B high expression compared to patients with FAM83B low expression (*p* < 0.001). Furthermore, the follow-up periods of the patients varied between 26.9 to 50.9 months (median 33.2 months) in high expression group. But there were few events in low expression group and the median time could not be counted. And the PFI durations were shorter in patients with FAM83B high expression compared to patients with FAM83B low expression (p < 0.001) (Fig. 1D–F).

### Protein levels of FAM83B and the correlation between the expression and clinicopathological features in LUAD

FAM83B was mainly located in the cytoplasm. We assessed the expression levels of FAM83B protein in 126 paraffin embedded primary LUAD samples. The positive rate of FAM83B was 23.80% (30/126) in tumor tissues, which was significantly higher than those in para-carcinoma tissues (*P* < 0.0001) (Fig. 2). Furthermore, we analyzed the correlation between the expression of FAM83B and clinical pathological characteristics of 126 primary LUAD patients. The results showed that the higher expression of FAM83B had a positive relation with lymph node metastasis (*p* = 0.048); On the contrary, there was no difference between FAM83B expression and patients' age, gender, tumor size, histological subtype or TNM stage (Table1).

**Table 1:** The relationship between the expression of FAM83B and the clinicopathological features in LUAD

	FAM83B expression			
Charactertistic	Total	High	Low	*P*
	*n* = 126	*n* = 30	*N* = 96	
Gender				
Female	66	13	53	0.888
Male	60	17	43	
Age(Y)				
< 65	88	18	70	0.178
> 65	38	12	26	
Smoking history				
Yes	50	18	38	0.968
No	76	12	58	
Clinical stage				
I-IIA	61	11	50	0.14
IIB-IV	65	19	46	
LN metastasis				
Yes	60	11	41	0.048
No	66	19	55	
Tumor differentiation				
Well/Moderate	82	20	62	0.835
Poor	44	10	34	

### Enrichment analysis of FAM83B gene co-expression network in LUAD

**Fig. 3:**
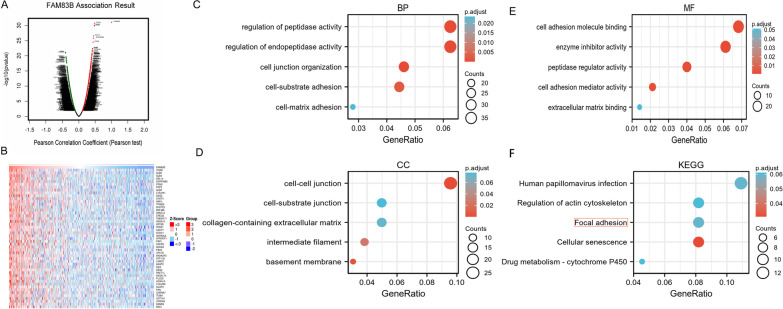
The enrichment analysis of FAM83B function in LUAD. **A**: The genes highly related to FAM83B were found in the LUAD cohort by Pearson test. **B**: The heatmap displays the top 50 genes positively associated with FAM83B in the LUAD cohort. **C**–**E**: Enrichment of CC、BP and MF for genes related to FAM83B, respectively. **F**: Gene terms related to FAM83B in Enrichment of Kyoto Encyclopedia of Genes and Genomes (KEGG)

To explore the biological significance of FAM83B in LUAD, we used the LinkedOmics database to analyze the FAM83B co-expression in LUAD. Figure 3A was the volcano map of co-expression genes, the results showed 4894 genes are positively related with FAM83B and 4266 genes are negatively correlated with FAM83B (both *p* < 0.05), statistically; And Fig. 3B was the heat map of the top 200 positively correlated genes. The detailed descriptions of related genes were shown in Additional file 4: Table 1.

The KEGG pathway database is the main database in KEGG [12]. R software package was used to perform GO and KEGG enrichment analysis of the top 200 co-expression genes of FAM83B. Under the condition of p.adj < 0.1, there are 65 biological process (GO-BP), 19 cellular components (GO-CC), 37 biological process (GO-MF) and 11 KEGG. The bubble chart shows the 15 pieces of BP, CC, MF and KEGG, respectively. GO function annotation shows that FAM83B co-expression are involved in cell–matrix adhesion, cell–cell junction, extracellular matrix binding (Fig. 3C–E). While KEGG pathway analysis showed that FAM83B co-expression are mainly related to the focal adhesion signaling pathway (Fig. 3F). Additional file 5: Table 2 summarized the GO and KEGG enrichment analysis details of FAM83B co-expression.

### FAM83B enhanced migration and invasion of LUAD cells in vitro

**Fig. 4:**
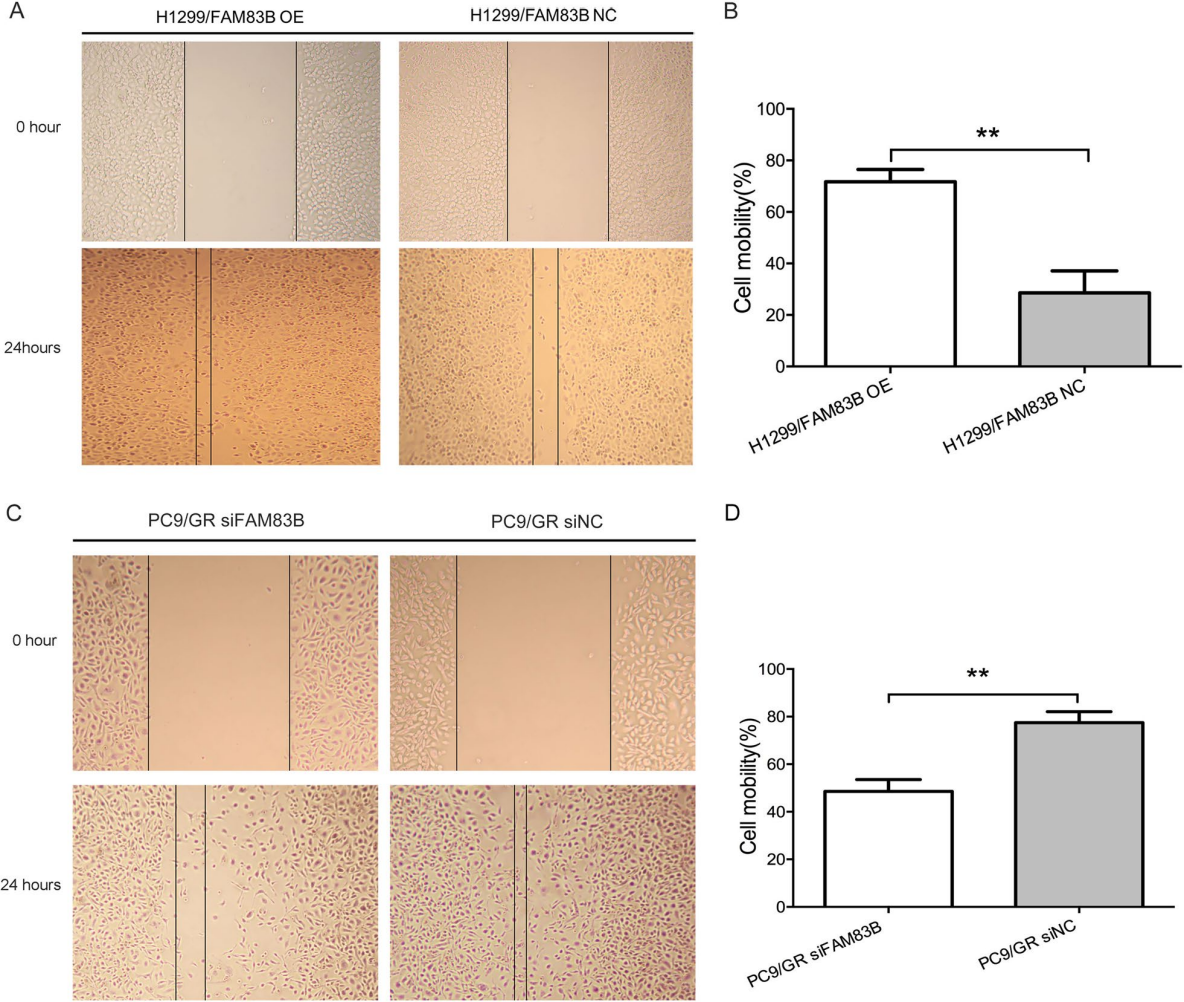
The effect of FAM83B overexpression on migration of LUAD. **A** and **B**: the migration rate of H1299/ FAM83B OE group compared with control group, * *P* < 0.05. **C** and **D**: the migration rate of PC9/GR FAM83B siRNA group compared with control group, * *P* < 0.05

**Fig. 5:**
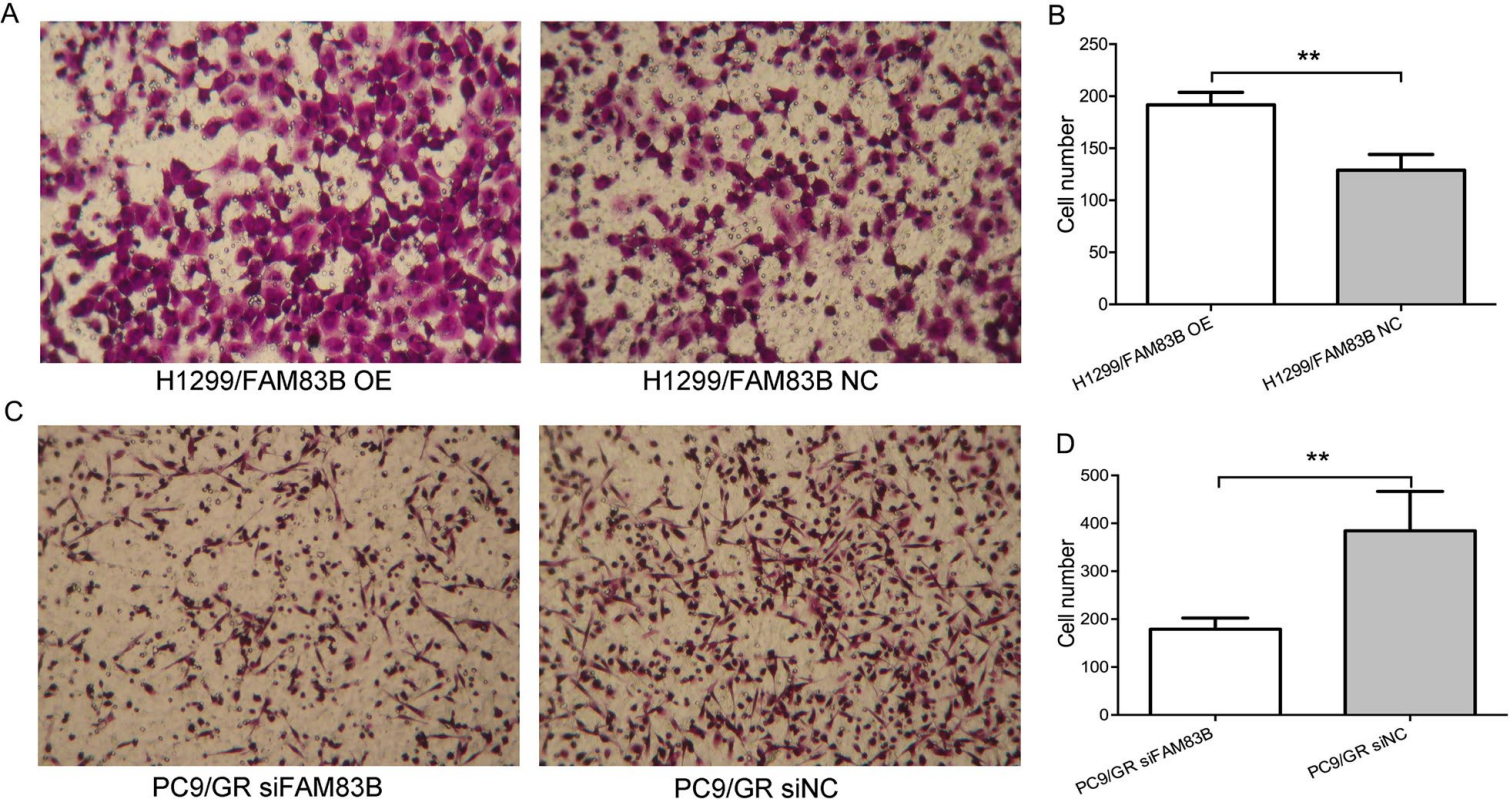
The correlation between the expression of FAM83B and the invasive capacity of LUAD. Compared with the parental cells, **A** and **B** showed the number of cells translocating the chamber in H1299/FAM83B OE group was ** *P* < 0.01*;* **C** and **D** showed the number of cells passing through the chamber in PC9/GR FAM83B siRNA group, * *P* < 0.05

The impact of FAM83B overexpression or silence on LUAD cell migration or invasion in vitro were detected by scratch assay and transwell assay, respectively. The scratch-wounding test displayed the cell migration was significantly faster in H1299/FAM83B OE group than that in H1299/FAM83B NC cells. While in PC9/GR FAM83B siRNA group, the result is just opposite (Fig. 4). As shown in Fig. 5, the cell number of H1299/FAM83B OE was about 24.5% higher in H1299/FAM83B OE than that in H1299/FAM83B NC; Conversely, knockdown of FAM83B gene maybe reduce the invasive capacity of PC9/GR FAM83B siRNA of about 50.3% compared with PC9/GR FAM83B siNC. Therefore, high level of FAM83B was able to promote the migration and invasiveness in LUAD cells.

### FAM83B promoted the tumorigenesis and capsule invasion in vivo

**Fig. 6:**
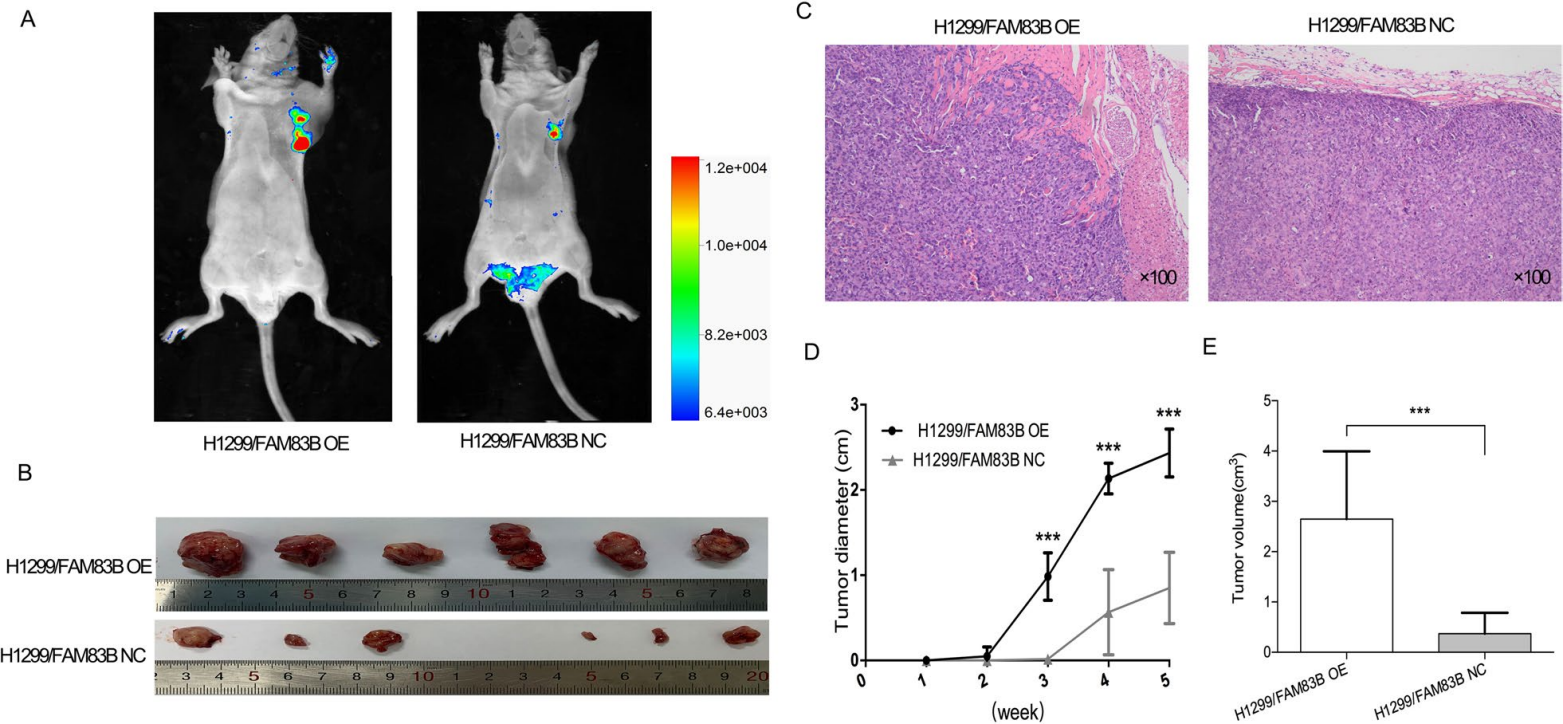
Tumorigenesis experiment in nude mice in vivo. The BALB/C nude mice were inoculated with H1299/FAM83B OE and H1299/FAM83B NC cells, respectively (*n* = 6/group); **A** and **B**: After 5 weeks, the tumors of H1299/FAM83B OE were significantly larger than those of H1299/FAM83B NC, ** *P* < 0.05; **C**: the results of H&E staining between H1299/FAM83B OE group and H1299/FAM83B NC group; **D**: the tumor growth curve measured every week after injection. **E**: The comparison of tumor volume between OE and NC groups, *** *P* < 0.01

To clarify the effect of FAM83B on LUAD oncogenesis in vivo, overexpression and negative control of H1299 cells were transplanted into 4-week nude mice. About 2 weeks after inoculation, the tumor could be touched subcutaneously in the transplantation location in the OE group; 5 weeks later, the tumor weight was more than 2 g in the OE group, and the experiment was terminated according to the principle of animal experiment. At 1, 2, 3, 4 and 5 weeks, the tumor diameter of H1299/FAM83B OE group was 0, 0, 0.05, 0.98, 2.13 and 2.42 cm, respectively; While that of the control group was 0, 0, 0, 0.02, 0.57 and 0.85 cm, respectively. From the third week, the mean volume of the tumors in H1299/FAM83B OE group was significantly larger than those in H1299/FAM83B NC group (all *p* < 0.001). Then the specimens were cut out, and the integrity of the tumor envelope was observed; Hematoxylin and eosin staining showed that the forefront of tumor infiltration in the OE group was irregular, suggesting the change of capsule invasion, while the surface of the NC group was regular (Fig. 6). The above results demonstrated that overexpression of FAM83B can promote the proliferation and invasion of lung adenocarcinoma in vivo.

### FAM83B regulated NF-κB translocation to induce the TIMP-1 expression via PI3K/AKT pathway in LUAD cells

**Fig. 7:**
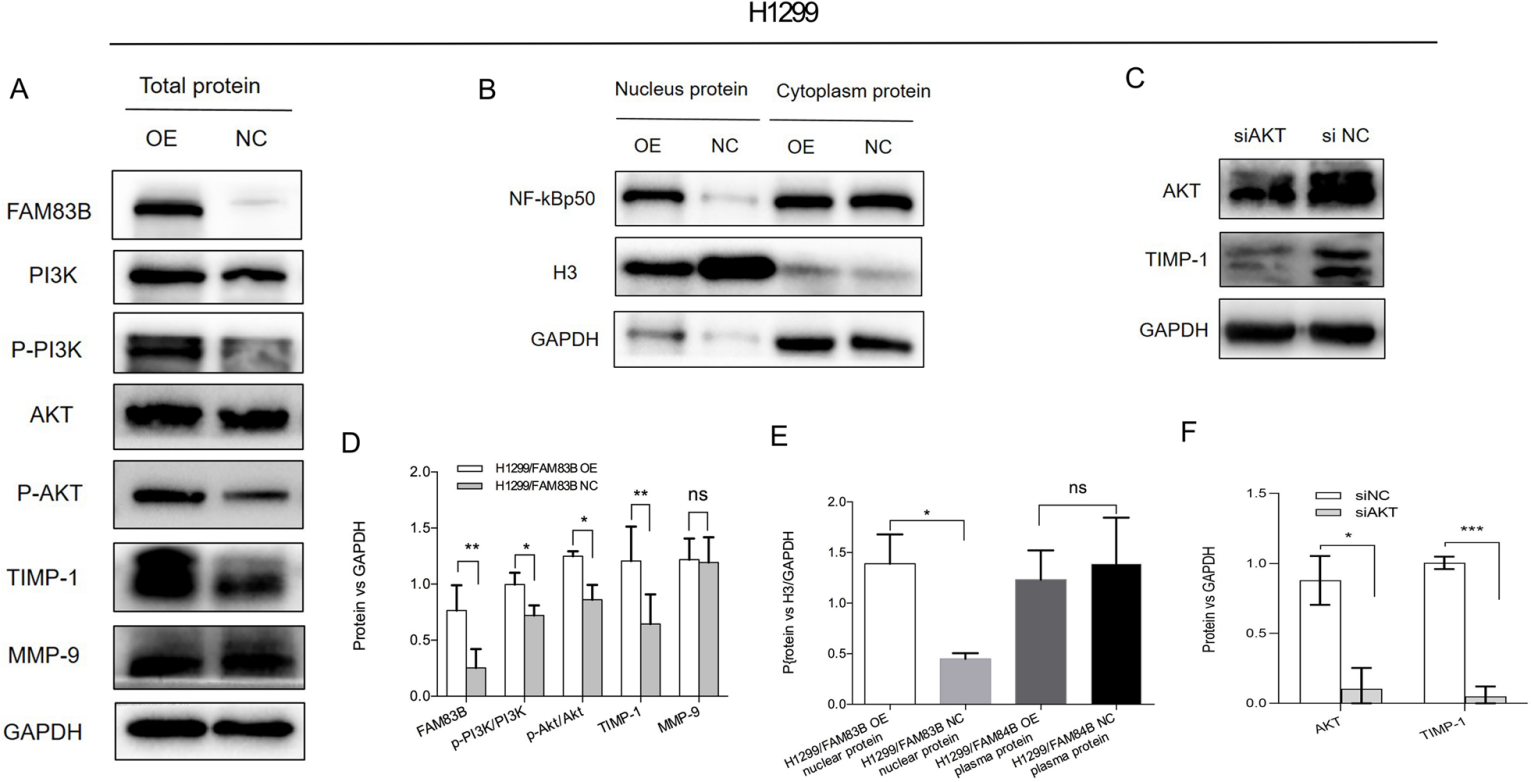
The effect of FAM83B on the levels of MMP9 and TIMP-1 by activating the PI3K/AKT/NF-κB signaling pathway in H1299 cells. The images of adequate length were not provided in the manuscript. **A**–**B**: Western blotting was performed to check the effects of FAM83B on the levels of MMP9 and TIMP-1, the activation of PI3K and AKT, as well as the distribution of NF-κB. **C**: The rescue test of PI3K/AKT pathway in H1299/FAM83B OE cells. The cells were treated with siAKT or siNC. Western blotting was performed to check the expression of TIMP-1. **E**–**F**: The data shown in the histogram are the mean ± SD of three independent experiments. *P < 0.05, **P < 0.01, ***P < 0.001

MMP-9/TIMP-1 imbalance is a common mechanism of malignant tumor invasion and metastasis, and PI3K/AKT/NF-κB pathway plays a key role in the process of lung cancer metastasis. In order to exclude the mechanism of FAM83B in the invasion and metastasis of LUAD and explore the possible signal pathway, western blotting was applied in H1299/FAM83B OE and H1299/FAM83B NC cells. The results manifested that there was no change of MMP-9 expression between the two groups (*p* > 0.05); While the level of TIMP-1 was one time higher in H1299/FAM83B OE cells than that in H1299/FAM83B NC group (*p* < 0.01). The relative levels of p-PI3K/PI3K and p-AKT/AKT were increased, compared with the control group (*P* < 0.05). Furthermore, in the H1299/FAM83B OE group, the amount of NF-κB protein translocating to the nucleus was significantly higher than that in the control group (Fig. 7). These results suggested that FAM83B might regulate the expression of TIMP-1 through activating the PI3K/AKT/NF-κB signaling pathway.

## Discussion

FAM83B is a member of FAM83 family and locates on human chromosome 6. It contains 1011 amino acids and the molecular weight is about 110kD. It has been confirmed that the expression of FAM83B mRNA and protein significantly increases in several types of carcinoma, such as breast cancer, ovarian cancer, cervical cancer, testicular cancer, thyroid cancer, bladder cancer and lymphoid cancer [13]. Okabe et al. found that the positive rate of FAM83B expression was 94.7%, the sensitivity was 94.5% and specificity was 85.3% in NSCLC; while the positive rate was only 14.7% in adenocarcinoma. So, they proposed that FAM83B was a reliable diagnostic marker for the lung squamous carcinoma, and its high expression was negatively correlated with the DSS of squamous carcinoma, but not with the OS [8].

Herein, we analyzed the expression of FAM83B mRNA in LUAD from TCGA database and FAM83B protein from CPTAC of UALCAN. The results showed that, compared with normal tissues, that FAM83B was over-expressed in 10 types of cancers including the LUAD; Moreover, the level of FAM83B was higher than that in normal control from CPTAC. The survival analysis of Kaplan Meier plot database showed that the OS, DSS and PFI of FAM83B high expression group were significantly lower than that of low expression group. Abnormal regulation of growth signal raised from the activation process of growth factor receptor or its downstream effector plays an important role in tumorigenesis and development. Cipriano et al. reported that FAM83B can specifically bind to c-raf of RAS, resulting in destroying the interaction between c-raf and 14–3-3, thus increasing the accumulation of c-raf in cell membrane and leading to the activation of MAPK as well as mTOR signal pathway in immortalized mammary epithelial cell lines. On the other hand, Sarah et al. found that the high expression level of FAM83B in 216 patients with primary LUAD was positively related to male dominance, wild-type EGFR status, lymph node involvement, pleural and vascular invasion and it could be regarded as a potential therapeutic target for LUAD with wild-type EGFR [7]. In this study, we first detected the expression of FAM83B protein by immunohistochemistry in paraffin samples of primary LUAD and found that the level of FAM83B was significantly higher than that in the normal lung tissues, and the results were consistent with that of RNA expression. Although the positive rate of FAM83B was only 23.8%, it was positively correlated with lymph node involvement, but not related with gender, age, smoking history, histological subtype or tumor stage. In our research, about 47.2% (61/129) of the cases were primary LUAD with the diameter no more than 3 cm and without lymph node metastasis, in another word, the cases are in IA stage, so the prognosis is relatively better. So, we didn't get the statistically significant follow-up results in this study. In the future, we shall continue the follow-up these cases, or increase the cases of the advanced LUAD for further analysis. In summary, the results suggest that high expression of FAM83B is apt to metastasize to lymph node and it may be a sign of poor prognosis for LUAD.

In order to investigate whether overexpression of FAM83B has the effect of promoting tumor invasion and metastasis in LUAD, the cell lines of H1299 with lower expression of FAM83B protein and mRNA and PC9 with higher expression of them were selected. In view of the drug-resistant cells have the stronger metastatic ability, we induce T790M mutation and established PC9/GR with gefitinib in PC9 cells. Then, we established the H1299 of FAM83B overexpression cell line (H1299/FAM83B OE) and the PC9/GR of FAM83B targeting silence cell line (PC9/GR/FAM83B siRNA). The phenotype research found the over expression of FAM83B can promote the migration and invasion of LUAD cells. The tumorigenesis experiment of nude mice results showed that the tumorigenesis in H1299/FAM83B OE was significantly quicker than that in the control group. Furthermore, compared with the control, the border of the tumor infiltrated the surrounding tissue irregularly and capsule invasion was easily to be observed in the OE group. Shen et al. found that inhibition of FAM83B could promote the expression of p21 protein, and then reduce the level of CDK4, CDK6 and cyclin D1, resulting in cell arrest in G0/G1 phase, but knockout FAM83B did not affect the invasion and apoptosis of pancreatic cancer cells [4]. Lin et al. showed that linc00324 could increase the stability of FAM83B by binding to human antigen R (HuR), thus promoting the proliferation and migration of cancer cells in gastric adenocarcinoma [5]. In LUAD, the high expression of FAM83B is related to lymph node metastasis and vascular invasion. These results suggested that the over expression of FAM83B might enhance the proliferation of LUAD and promote its migration and invasion, but the possible mechanism has not been clarified. Now, no report on the functional enrichment analysis of FAM83B co-expression in LUAD has been published. In this research, we analyze the FAM83B co-expression gene of LUAD in LinkedOmics database. GO and KEGG functional enrichment analysis of the top 200 genes related to FAM83B showed that the FAM83B co-expression is mainly related to the structural constituent of cell–matrix adhesion, intermediate filament, cell–cell junction and extracellular matrix binding; While the KEGG pathway analysis suggested that the FAM83B co-expression was mainly related to the focal adhesion signaling pathway. The results of these functions and pathways suggest that FAM83B may affect extracellular matrix (ECM) and focal adhesion and participate in the migration or metastasis of LUAD.

More than 90% of cancer-related deaths are caused by relapse or metastasis of the tumors [14]. The tumor cells penetrate the basement membrane and degrade ECM in the process of invasion and metastasis. The ECM is composed of hundreds of molecules, including MMPs and TIMPs. There are many kinds of proteolytic enzyme involved in ECM degradation and matrix metalloproteinases (MMPs) play an important role in this process. MMPs belong to a family of neutral proteases, which can hydrolyze almost all protein components in extracellular matrix. MMP-9 is an important proteolytic enzyme of MMPs, which is closely related to the invasion and metastasis of a variety of malignant tumors [15]. It is reported that MMP-9 is related to invasion, metastasis and prognosis of lung cancer, and those with high expression of it are prone to metastasis [16]. But it is not clear whether the role of FAM83B in invasion and metastasis is related to the promotion of MMP-9 expression and secretion. To our surprise, compared with the control group, the expression of MMP-9 in H1299/FAM83B OE cells did not increase.

As we known, MMPs and their inhibitors of tissue inhibitor of metalloproteinases (TIMPs) which can interact with MMPs and affect the progression of cancer are in a dynamic balance during the process of ECM degradation and reconstruction, so TIMPs maybe also play an equally important role in the process. In this study, the expression of TIMP-1 in H1299/FAM83B OE cells were also detected. The results showed that compared with the control group, the expression of TIMP-1 was twice that of the control group. TIMP-1, as an inhibitor of MMP-9, binds specifically to the end of carboxyl group in the catalytic region of proenzyme or activated enzyme of MMP-9 to form a complex, thus specifically inhibit the activity of MMP-9. The role of TIMP-1 in tumor growth, invasion and metastasis remains controversial. MMPs and TIMPs play an important role in several stages of tumorigenesis [17–19]. Initially, MMP was considered to be involved in the beginning of metastasis due to the disintegration of the physical barrier corresponding to ECM and basement membrane collagen; TIMPs can inhibit tumor invasion and metastasis by inhibiting the proteolytic activity of MMPs [20, 21]; However, several studies have shown that TIMP-1 is a multifunctional protein with the effect in progression and metastasis of carcinoma and the destruction of the balance between MMPs and TIMPs is related to the progression of cancer [22]. In addition to the function of inhibiting MMP, TIMP-1 can also promote cell growth and proliferation, inhibit apoptosis, and participate in the regulation of angiogenesis. And the high level of TIMP-1 is related to the poor prognosis or tumor progression of a variety of malignant tumors [23].

In the past, TIMP-1 was considered to be a negative regulator of tumor metastasis, but more and more studies have found that high expression of TIMP-1 in NSCLC, colorectal cancer, breast cancer, gastric cancer, prostate cancer, endometrial cancer and so on often with poor prognosis [24]. For example, LUAD patients with high expression of TIMP-1 are prone to lymph node or distant metastasis. The results of the cell showed that the down-regulation of TIMP-1 inhibited the migration, invasion and metastasis of LUAD cells [25]; While the high level of TIMP-1 in serum or plasma of colon cancer patients is negatively related to the survival of patients. The role of TIMP-1 can be divided into MMPs dependent and MMPs independent. The former is achieved by interacting with MMPs, up regulating the expression of TIMPs in tumor cells to inhibit tumor invasion and metastasis; the latter can promote tumor cell proliferation, survival and induce neovascularization through receptor-mediated signal pathway. TIMP-1 is not only a protease inhibitor, but also can promote tumor cell proliferation, survival and induce angiogenesis. In other words, it may also play an important role in tumor metastasis [26, 27]. So, it is speculated that TIMP-1 may play an effect based on MMP-9 independent pathway in our experiment.

Furthermore, it was found that there were specific binding sites of positive regulatory elements of nuclear factor kappa B (NF-κB) and ATF-2, which located the upstream the promoter region of TIMP-1 in the study of astrocytoma [28]. Further analysis of the promoter region from upstream 2000 bp to downstream50 bp of TIMP-1 gene by using Promo database showed that there was a positive binding sequence of NF-κB among region, indicating that there may be a binding site of NF-κB in the promoter region of TIMP-1.

Continuous acquisition of growth stimulating signals is one of the important characters of lung adenocarcinoma and receptor tyrosine kinases (RTK) and phosphatidylinositol-3-kinase (PI3K) signaling pathways are important driving factors. PI3K/serine threonine protein kinase (AKT) signaling pathway not only takes part in the tumor survival and proliferation. RTK receptor and PI3K/ AKT signal can be abnormally activated without the presence of growth factors [29]. AKT is downstream of PI3K and upstream of NF-κB. The activation of PI3K promotes AKT accumulated to the cell membrane and phosphorylates threonine 308 (thr308) or serine 473 (ser473) of AKT. The phosphorylated AKT is separated from the cell membrane to activate IKK, which leads to the degradation of inhibitor of nuclear factor κB (IκB) which is an inhibitor of NF-κB. Thus, the NF-κB releases from the cytoplasm to translocate to nuclear, then activates the different target genes and results in the development, metastasis, invasion and angiogenesis of tumor [30]. The activation of PI3K/AKT pathway has been observed in most non-small cell lung cancer, which promotes proliferation, migration, invasion and drug resistance [31]; In view of the fact that FAM83B can be involved in activating PI3K/AKT/mTOR signal pathway, we detected the key signal molecules of PI3K/AKT pathway in H1299/FAM83B OE cells. The results showed that overexpression of FAM83B significantly increased PI3K and AKT phosphorylation. Therefore, it is suggested that FAM83B may increase the expression of TIMP-1 by activating PI3K/AKT/NF-κB signaling pathway, while TIMP-1 may play its role in promoting the migration and invasion of LUAD through non MMP-9 dependent pathway. It has been found that overexpression of TIMP-1 leads to the decrease of E-cadherin and the increase of vimentin level in MCF-10A cells, leading to epithelial mesenchymal transition (EMT) of breast epithelial cells. In addition, TIMP-1 can interact with the fourth transmembrane protein CD63, and then activate signal molecules such as FAK, PI3K, AKT and MAPK, suggesting that TIMP-1 may play a variety of roles in the occurrence and development of tumors [32–34].

In conclusion, our study found that the expression of FAM83B in primary LUAD was higher than that in normal pulmonary tissue and the high expression of FAM83B was related to lymph node metastasis of patients; and high expression is a marker for poor prognosis. In vitro and in vivo experiments suggest that FAM83B may regulate the expression of TIMP-1 through PI3K/AKT/NF-κB pathway, and then promote the proliferation, migration and invasion of LUAD.

## Supplementary Information

**Additional file 1.** Raw data of western blot.

**Additional file 2 Fig S1** Establishment of LUAD cell line.** A–C**: The expression of FAM83B protein and mRNA in H1299/OE and H1299/NC cell lines, respectively. **D–E**: The expression of FAM83B protein and mRNA in PC9/GR siNC and PC9/GR si FAM83B cell lines, respectively.

**Additional file 3. Fig. S2** Detection of interference efficiency of siRNA. **A** and **B**: The results of western blotting showed that compared with the level of siNC group, the interference efficiency of si-2 and si-3 were higher. **C**: The interference efficiency of FAM83B siRNA was detected by qPCR.

**Additional file 4. Supplementary Table 1.** The related genes of FAM83B resulted from the LinkedOmics database.

**Additional file 5. Supplementary Table 2.** The GO and KEGG enrichment analysis of the top 200 co-expression genes of FAM83B.

## Data Availability

The mRNA sequencing results of in human carcinomas and LUAD from TCGA database were analyzed in website of xiantao.love (https://www.xiantao.love/). LinkedOmics database was used to analyze the FAM83B co-expression in LUAD (http://linkedomics.org/login.php).
