# Supplementary figures and images for: FAM83B promotes the invasion of primary lung adenocarcinoma via PI3K/AKT/NF-κB pathway
Source: BMC Pulm Med. 2023 Jan 23;23:32. doi: 10.1186/s12890-022-02303-5 (PMC9872310; doi:10.1186/s12890-022-02303-5)

Figure 7A

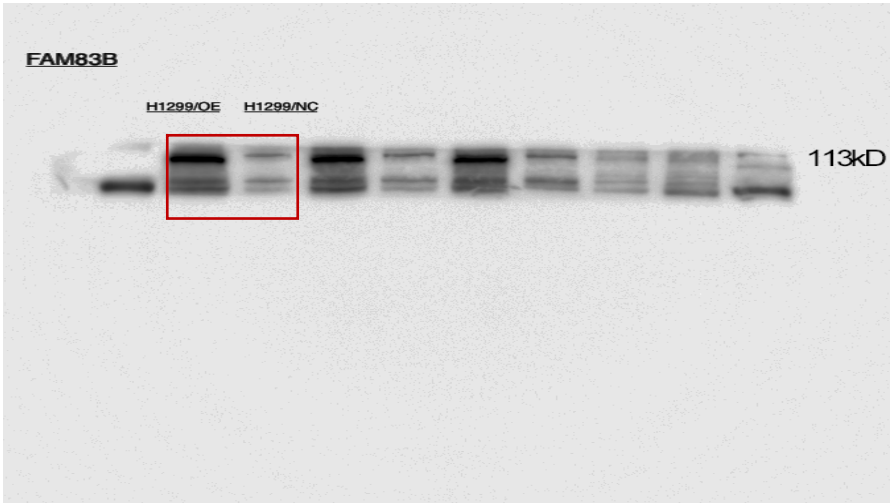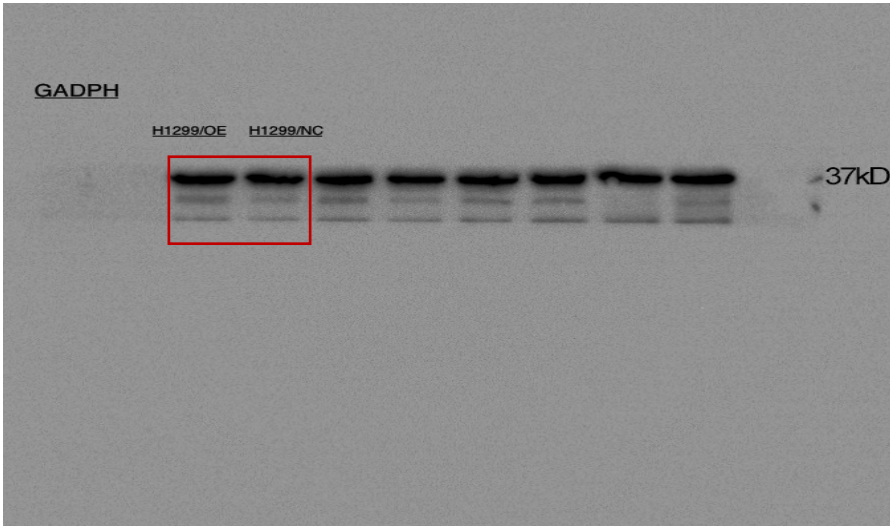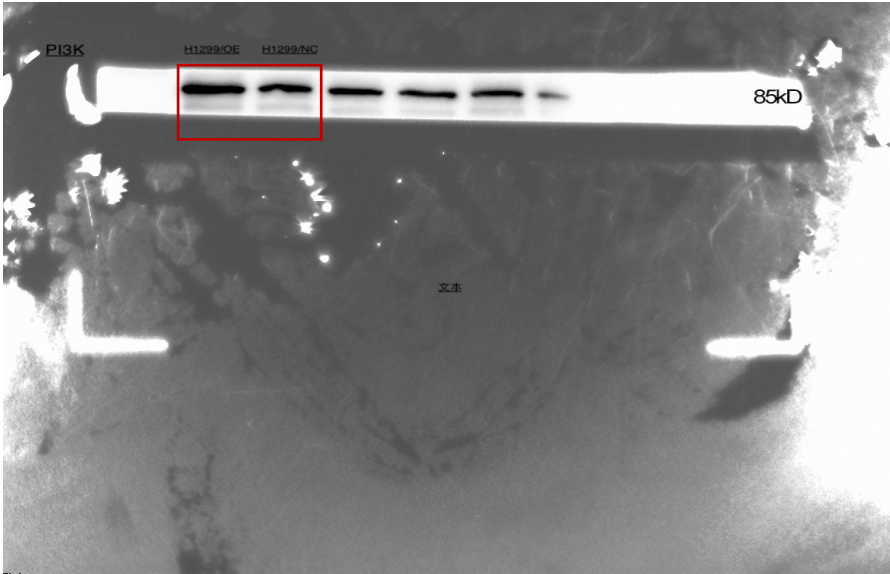

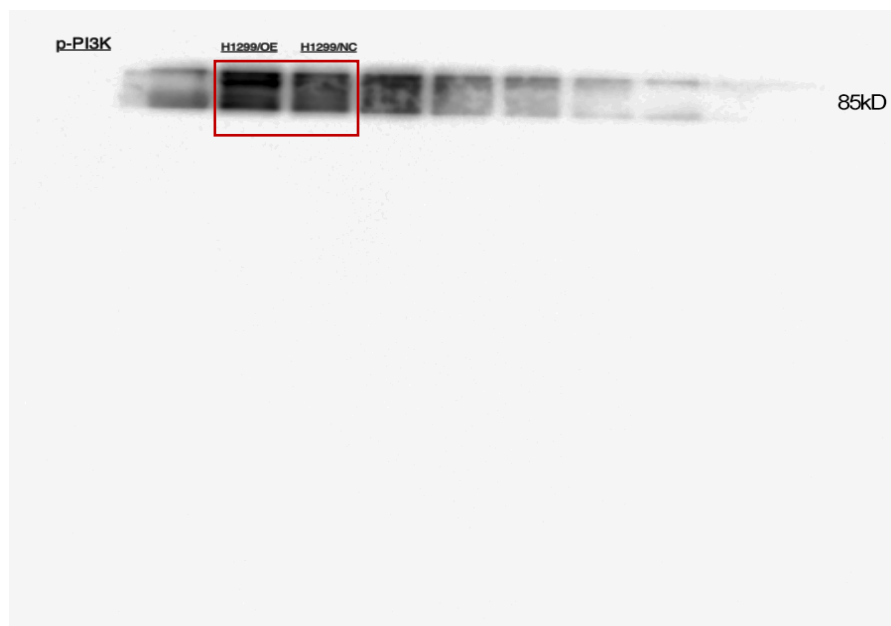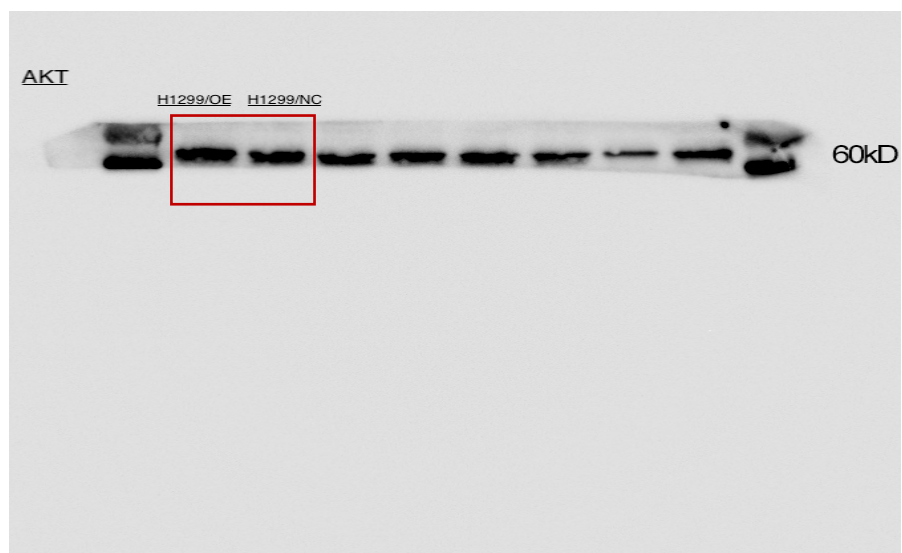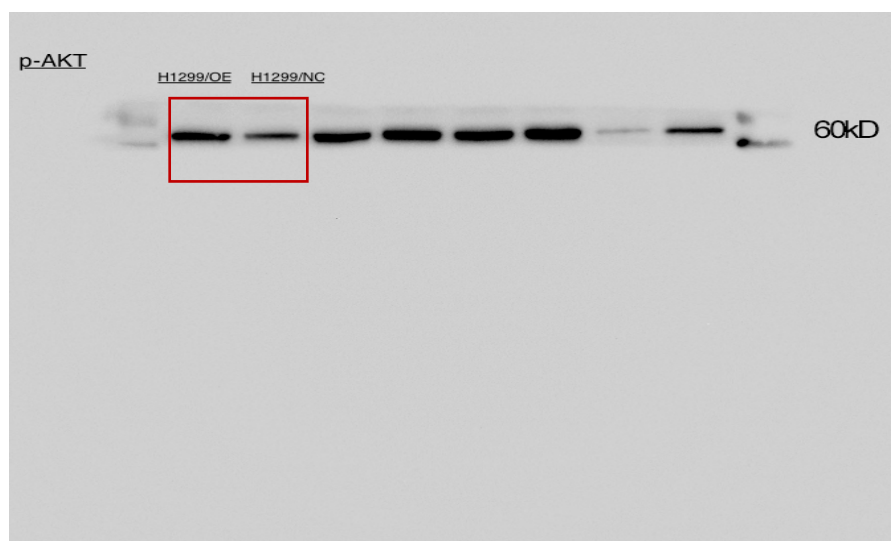

GADPH of PI3K, p-PI3K, AKT, p-AKT

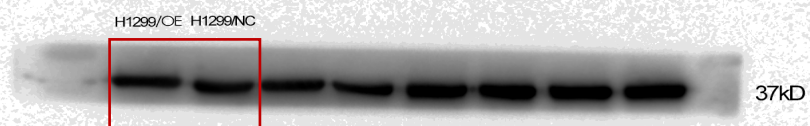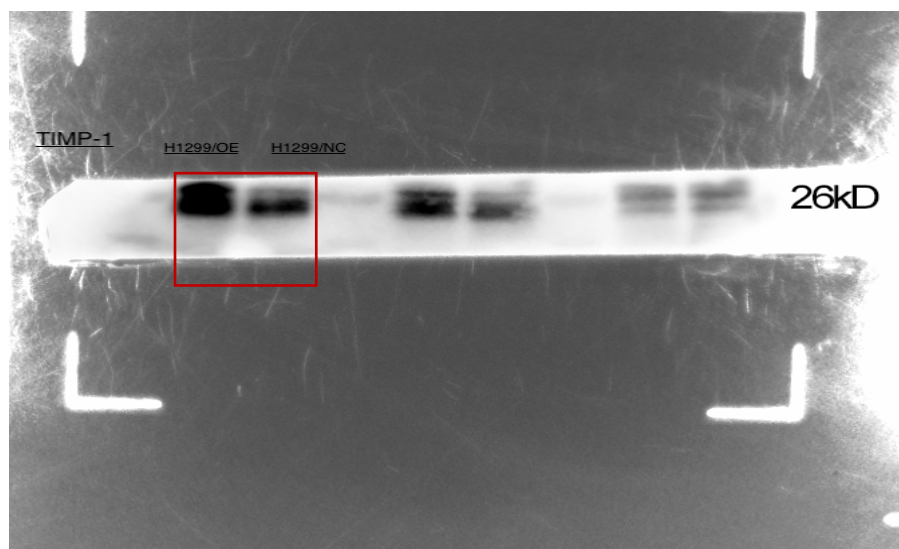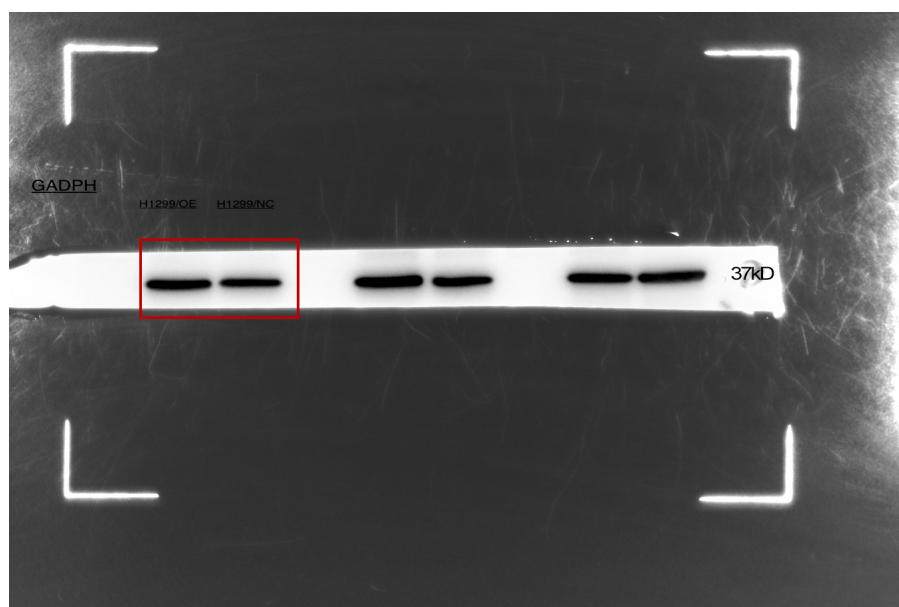

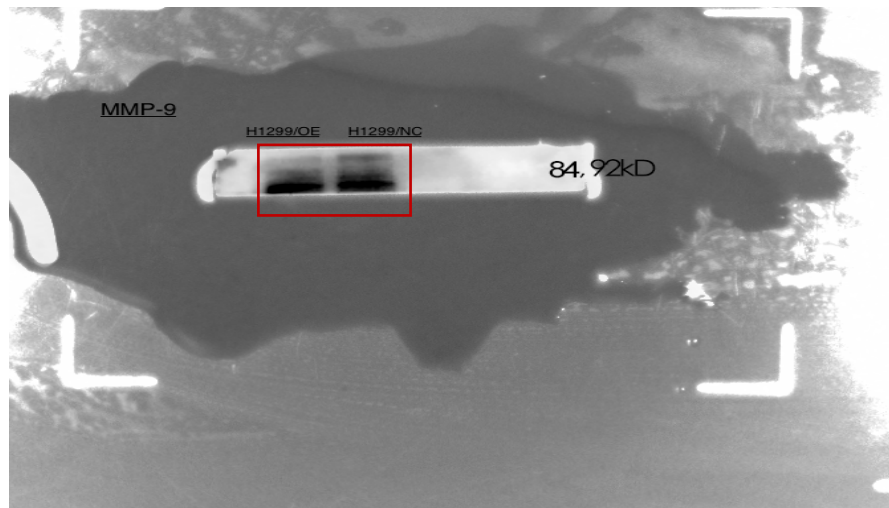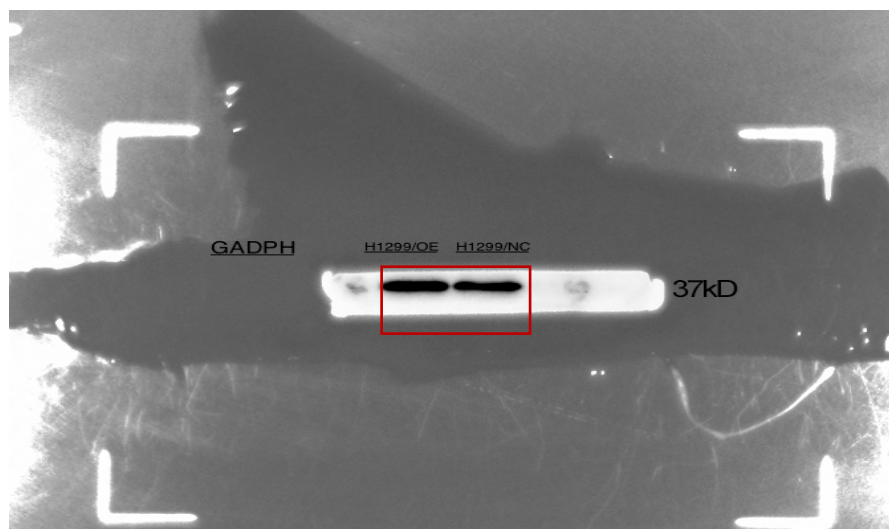

Figure 7B

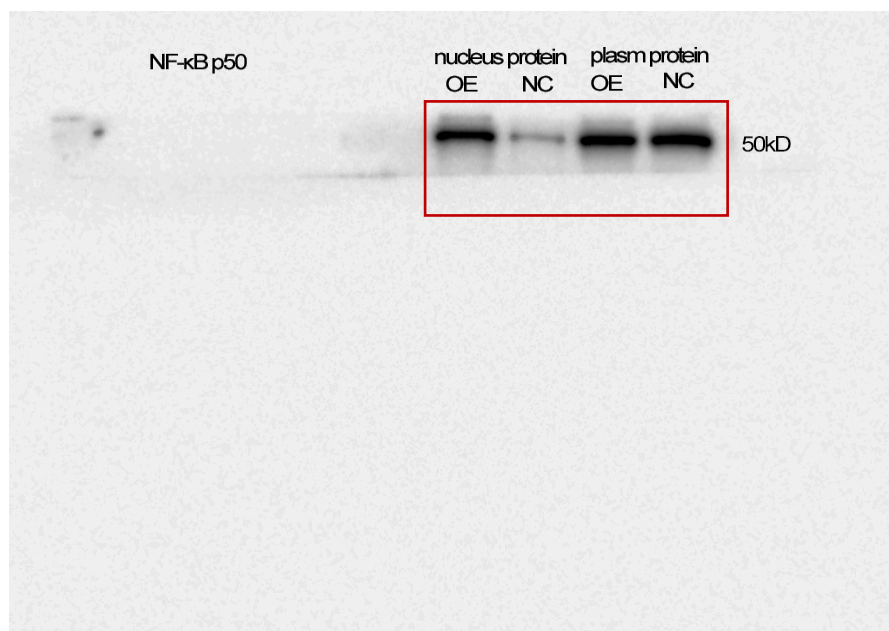

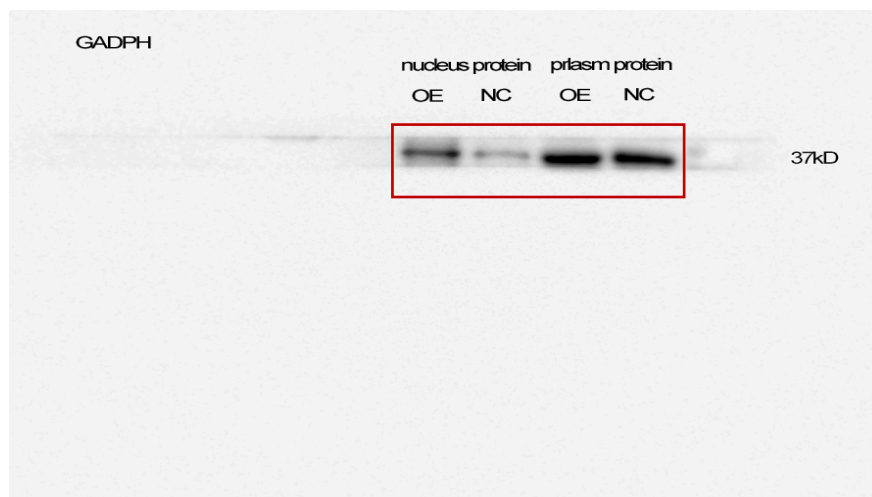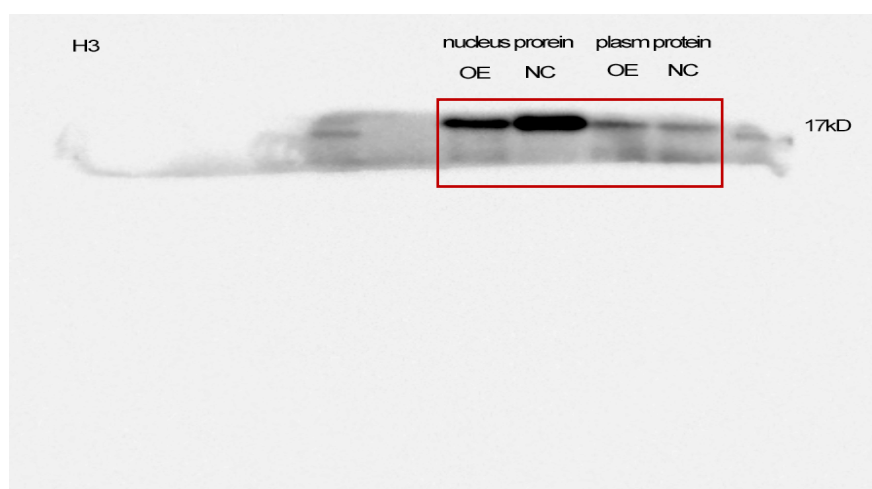

Figure 7C

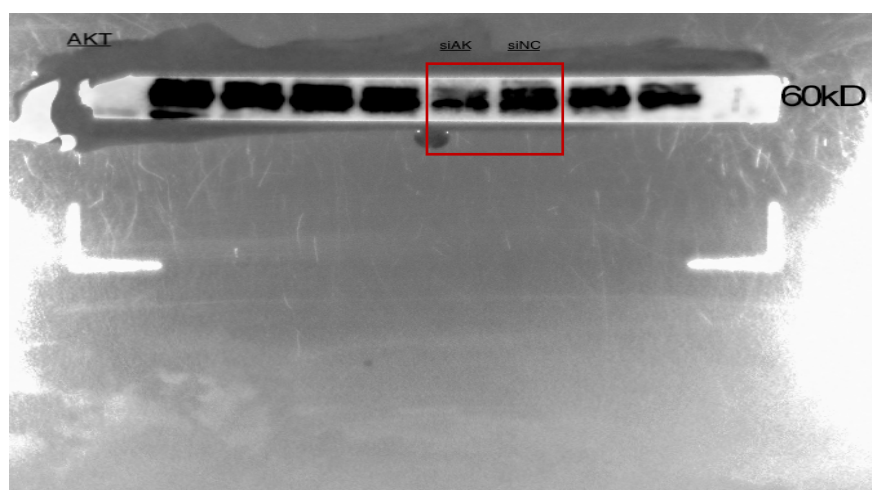

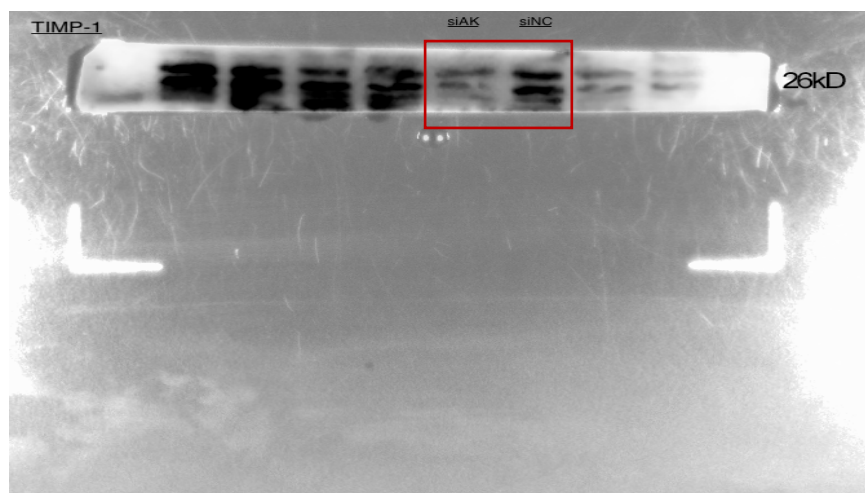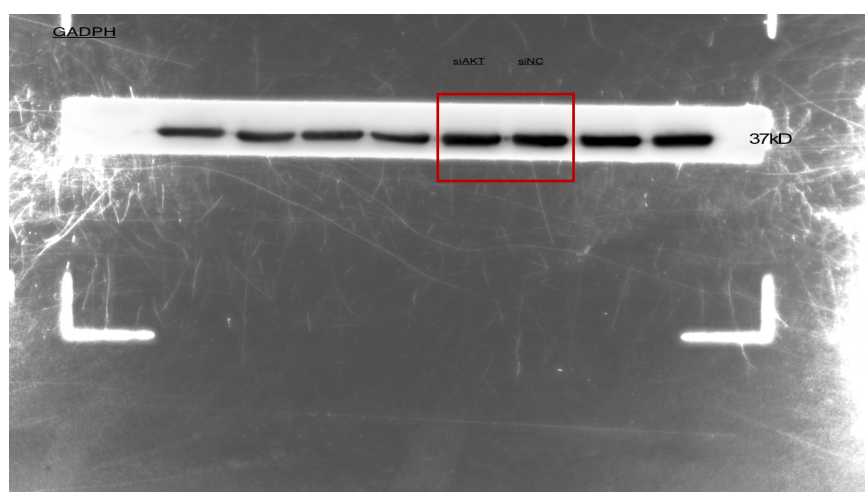

Supplement: Supplementary file 1 — Additional file 1. Raw data of western blot. [file 12890_2022_2303_MOESM1_ESM.pdf]

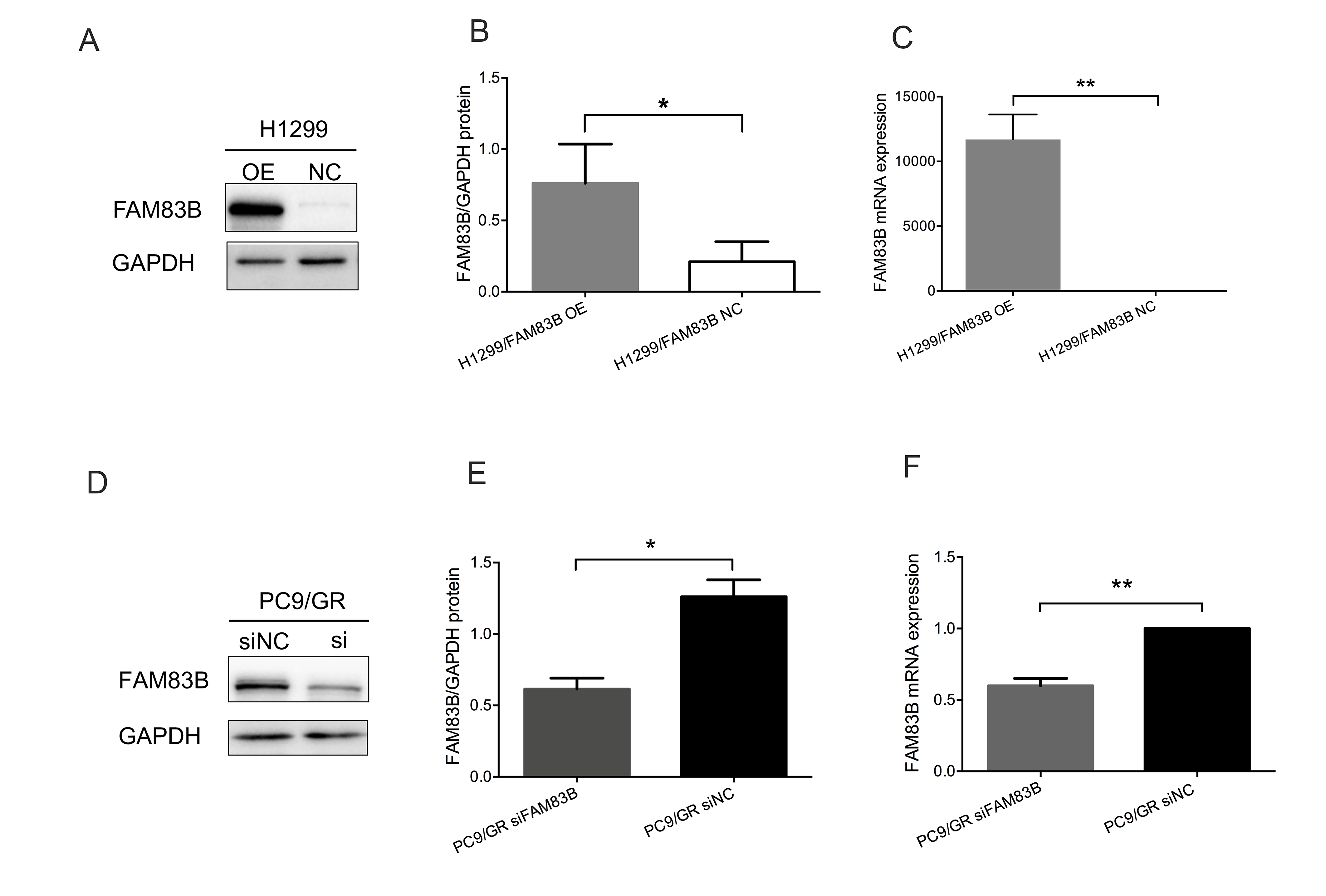

Supplement: Supplementary file 2 — Additional file 2 Fig S1 Establishment of LUAD cell line. A–C: The expression of FAM83B protein and mRNA in H1299/OE and H1299/NC cell lines, respectively. D–E: The expression of FAM83B protein and mRNA in PC9/GR siNC and PC9/GR si FAM83B cell lines, respectively. [file 12890_2022_2303_MOESM2_ESM.tif]

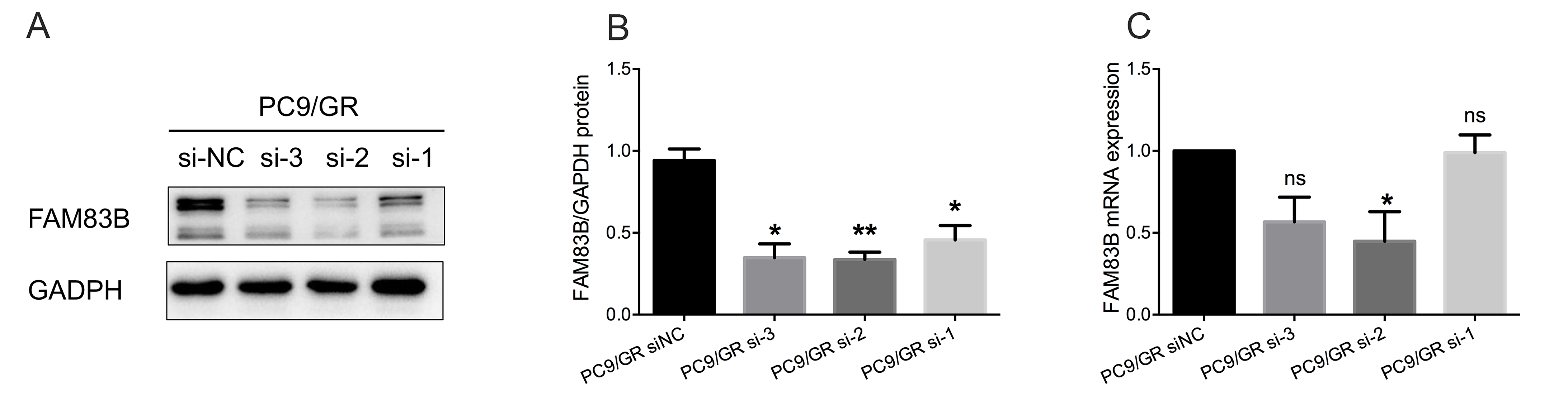

Supplement: Supplementary file 3 — Additional file 3. Fig. S2 Detection of interference efficiency of siRNA. A and B: The results of western blotting showed that compared with the level of siNC group, the interference efficiency of si-2 and si-3 were higher. C: The interference efficiency of FAM83B siRNA was detected by qPCR. [file 12890_2022_2303_MOESM3_ESM.tif]
